# Clinical application and institutional governance of foods for special medical purposes in medical institutions of eastern coastal China: a cross-sectional study

**DOI:** 10.3389/fnut.2026.1775765

**Published:** 2026-03-24

**Authors:** Qingqing Deng, Wanli Pu, Ting Zhu, Jiayao Yang, Junxuan Chen, Xiaosu Chen, Qing Zhao, Yue Xu, Shu-an Wang, Fangjun Chen, Xiaotian Chen

**Affiliations:** 1 Department of Clinical Nutrition, Nanjing Drum Tower Hospital, The Affiliated Drum Tower Hospital Clinical College of Xuzhou Medical University, Nanjing, Jiangsu, China; 2 Department of Oncology, Yunyang Hospital of Chongqing University of Traditional Chinese Medicine, Chongqing, China; 3 Department of Clinical Nutrition, Children's Hospital of Nanjing Medical University, Nanjing, Jiangsu, China; 4 Department of Clinical Nutrition, The Affiliated Hospital of Xuzhou Medical University, Xuzhou, Jiangsu, China; 5 China Hospital Reform and Development Research Institute of Nanjing University, Nanjing Drum Tower Hospital, Nanjing, Jiangsu, China; 6 Department of Oncology, Nanjing Drum Tower Hospital, Affiliated Hospital of Medical School, Nanjing University, Nanjing, Jiangsu, China

**Keywords:** clinical nutrition, FSMP, health policy, hospital management, medical insurance

## Abstract

**Introduction:**

Despite the established clinical value of Foods for Special Medical Purposes (FSMP) in improving patient outcomes, their integration into the Chinese healthcare system remains highly uneven. Using the economically developed Jiangsu Province as a representative model, this study aims to systematically evaluate the current status of FSMP clinical application and management, identify key systemic barriers, and provide empirical evidence to facilitate standardized management and policy optimization at both regional and national levels.

**Methods:**

A cross-sectional survey was conducted in October 2025. A stratified sampling strategy was employed to invite 115 medical institutions across Jiangsu Province. A total of 94 valid questionnaires were recovered, yielding an effective response rate of 81.7%. The structured questionnaire collected multidimensional data, including hospital characteristics, FSMP accessibility, internal management protocols, clinical usage, monitoring systems, insurance reimbursement, and staff cognition. Data were analyzed using descriptive statistics and Chi-square tests.

**Results:**

While the overall penetration rate of FSMP in surveyed hospitals was high (78.7%), standardization in management lagged significantly. Only 43.6% of hospitals had established an FSMP Management Committee. Although the variety of FSMP products available (median: 4) was comparable to that of “drug-coded” enteral nutrition preparations (median: 5), internal circulation pathways were fragmented, involving multiple departments such as nutrition and logistics. Payment barriers were prominent: only 2.1% of hospitals reported FSMP coverage by medical insurance, while 29.8% categorized costs as “meal fees.” Medical staff exhibited a dichotomy in cognition, characterized by high “product recognition” (e.g., 82.1% acknowledged taste advantages) but “insufficient clinical confidence” (e.g., 89.4% expressed concern over lack of reimbursement). Subgroup analysis revealed significant heterogeneity: economically developed regions (Southern Jiangsu) and tertiary public hospitals demonstrated significantly superior management and cognition levels (*p* < 0.05).

**Conclusion:**

The core issue is that the under-institutionalized and poorly regulated nature of FSMP is incompatible with China’s highly structured and protocol-driven medical management system. The sector is currently at a critical transition from “product accessibility” to “standardized management.” Future reforms must shift from “fragmented management” to “systematic governance” by clarifying the medical attributes of FSMP, establishing independent payment pathways, and strengthening information systems to overcome current impediments.

## 1 Introduction

Foods for Special Medical Purposes (FSMP) are specially processed and formulated to meet the specific nutritional or dietary requirements of individuals suffering from restricted food intake, digestive or absorption disorders, metabolic imbalances, or specific disease states (1). As a cornerstone of clinical nutrition therapy, FSMP has been globally proven to play a critical role in improving patient nutritional status, reducing the risk of complications, shortening hospital stays, and enhancing overall clinical outcomes (2, 3). Compared to traditional “drug-coded” enteral nutrition preparations, FSMP offers a broader range of product categories and formula designs that more precisely align with the metabolic characteristics and nutritional demands of specific diseases (4).

From an international perspective, the regulatory models for Foods for Special Medical Purposes vary significantly across countries, exhibiting a pronounced institutional dependency (5). The Codex Alimentarius Commission (CAC) issued the standard for the labeling of FSMP as early as 1991, laying the foundation for global trade. The European Union classifies FSMP into three categories pursuant to Regulation (EU) No 609/2013 and manages them through a post-market notification system (6). In the United States, FSMP are categorized as “medical food” and regulated by the FDA; they are exempt from pre-market approval but require compliance with Generally Recognized as Safe (GRAS) provisions. Japan, under the Health Promotion Act, implements a stringent pre-market approval system. Australia and New Zealand regulate FSMP under Standard 2.9.5 of the Australia New Zealand Food Standards Code, with no pre-market review requirement (7). These regulatory divergences reflect different underlying regulatory philosophies: Western countries emphasize post-market surveillance, whereas China and Japan prioritize pre-market validation. Despite the rapid growth of the global FSMP market, a common challenge persists: how to effectively translate these products into clinically accessible, controllable, and reimbursable therapeutic tools (8).

Following the official definition and categorization of FSMP by China’s national health authorities in 2013, the sector has entered a phase of rapid regulatory development. Landmark regulations, including the Administrative Measures for the Registration of Foods for Special Medical Purposes, have been implemented (5). These regulations mandate comprehensive and strict oversight across the entire product lifecycle—from R&D and clinical trials to registration, production, and sales—setting a significantly higher regulatory bar than that for ordinary foods (1). Despite the high-level attention and cautious approach from the state, significant gaps persist in the management and clinical application of FSMP at the institutional level. This contrast is evident in the fact that fewer than a quarter of medical institutions have set up dedicated FSMP management committees (9). Statistics indicate that over 60% of reported adverse reactions to FSMP can be linked to irrational clinical use, including inappropriate indications and improper administration methods (10). Furthermore, this is coupled with imbalanced clinical practices: only about one-third of hospitalized patients identified with nutritional risks actually receive nutritional support, while some patients without clear risks undergo unnecessary interventions (11). While these findings underscore the pressing need to enhance the scientific and standardized application of FSMP, few studies have moved beyond descriptive accounts to investigate the underlying causes or propose systemic solutions.

Recent years have witnessed a paradigm shift in FSMP research focus, from product development and efficacy verification (12–16) toward exploring clinical management pathways and implementation strategies. Standardized nutritional support therapy should adhere to the established protocol of “screening, assessment, diagnosis, intervention, and monitoring” (17). In 2021, a preliminary in-hospital FSMP management pathway was proposed for the first time in China, delineating specific roles for physicians, nurses, and dietitians across the processes of prescription, verification, review, preparation, and administration (18). This was followed by the release of the Expert Consensus on the Clinical Application of Foods for Special Medical Purposes, which provided more actionable professional guidance for standardizing FSMP use in China (19). Despite these advancements in policy and guidance, a fundamental question remains: why does on-the-ground management within hospitals remain chaotic?

Existing literature lacks a unified analytical framework capable of integrating multidimensional challenges such as management, payment, and cognition. This study argues that the core dilemma of FSMP in China stems from its dual regulatory identity: it is regulated as a food product under the Food Safety Law, yet functions as a medical intervention requiring clinical guidance, all within an under-institutionalized framework. Therefore, based in Jiangsu Province—a region with a developed economy and abundant medical resources—this study conducted a cross-sectional survey to achieve the following objectives: (1) to quantitatively evaluate the current status of FSMP accessibility and management; (2) to deeply analyze the core mechanisms underlying the major problems encountered in current FSMP processes; and (3) to uncover the heterogeneity across hospitals of different ownership models and tiers through subgroup analysis.

## 2 Participants and methods

### 2.1 Participants and sampling methods

An electronic questionnaire survey was conducted between September and October 2025 across 115 medical institutions in Jiangsu Province. A stratified random sampling method was employed, in which hospitals were stratified based on hospital grade (tertiary vs. secondary) and geographic region (Southern Jiangsu vs. Northern Jiangsu), with random selection performed within each stratum. A total of 94 valid questionnaires were retrieved, yielding an effective response rate of 81.7% (94/115). The survey covered all regions within Jiangsu Province, with the exception of Suqian City (see Table 1). The inclusion criterion was defined as the presence of either an independent Clinical Nutrition department or a comparable functional department.

**Table 1:** Baseline characteristics of the 94 participating hospitals.

Characteristics	Category	Number of hospitals (n)	Composition ratio (%)
Hospital grade	Grade III A-Class Hospital	54	57.4
	Grade III, Class B	19	20.2
	Grade II A-Class Hospital	8	8.5
	Other	13	13.8
Hospital category	General Hospital	73	77.7
	Specialty Hospital	20	21.3
	Traditional Chinese Medicine Hospital	1	1.1
The area where one is located	The southern region of Jiangsu Province	44	46.8
	The northern region of Jiangsu Province	50	53.2
Department of nutrition	Belonging to clinical field	31	33.0
	Under the jurisdiction of the medical technology department	57	60.6
	Under the logistics department	3	3.2
Hospital type	Other	3	3.2
	Public hospital	86	91.5
	Private hospital	8	8.5
Number of beds	≤500	7	7.5
	500–2000	72	76.6
	>2000	15	16.0
Is FSMP provided?	Yes	74	78.7
	No	20	21.3

### 2.2 Survey instrument and content

A self-designed questionnaire titled “Survey on the Status of Clinical Application and Management of FSMP in Medical Institutions” served as the primary data collection instrument. The questionnaire was developed following an extensive literature review and was based on the core content of the Expert Consensus on the Clinical Application of Foods for Special Medical Purposes (2021 Edition) (19). Subsequently, experts from the fields of clinical nutrition, hospital management, and health policy were invited to validate and revise the questionnaire’s dimensions and items, ensuring high content validity.

### 2.3 Data collection and quality control

Questionnaires were administered to the directors of clinical nutrition departments or their designated delegates who possessed comprehensive oversight of FSMP utilization within their units. This measure was implemented to ensure the authenticity, accuracy, and completeness of the collected data.

### 2.4 Statistical analysis

All statistical analyses were performed using IBM SPSS Statistics (Version 27.0). Categorical variables were described as frequencies and percentages. The normality of continuous variables was assessed using the Shapiro–Wilk test. Data are presented as mean ± standard deviation (SD) if normally distributed, or as median and interquartile range (IQR) otherwise. Comparisons across subgroups (e.g., by hospital grade or region) regarding management and clinical practices were conducted using the Chi-square test or Fisher’s exact test (the latter was utilized when more than 20% of cells had an expected frequency <5). As the study aimed to describe the current status and identify issues, the analysis was primarily descriptive and focused on simple inter-group comparisons; no complex multivariate models were constructed. All tests were two-sided, with a *p*-value < 0.05 considered statistically significant.

## 3 Results

### 3.1 Basic characteristics

A total of 94 medical institutions in Jiangsu Province were included in this study. The sample comprised 54 (57.4%) Grade III Level A hospitals, 19 (20.2%) Grade III Level B hospitals, 8 (8.5%) Grade II Level A hospitals, and 13 (13.8%) other institutions. In terms of hospital type, 73 (77.7%) were general hospitals, 20 (21.3%) were specialized hospitals, and 1 (1.1%) was a Traditional Chinese Medicine (TCM) hospital. Geographically, 44 hospitals (46.8%) were located in Southern Jiangsu, while 50 (53.2%) were in Northern Jiangsu. Regarding ownership, 86 (91.5%) were public hospitals and 8 (8.5%) were private hospitals. The median number of open beds was 1,015 (Q1 = 728, Q3 = 1,663). In 60.6% of the hospitals, the Department of Clinical Nutrition was administratively affiliated with the medical technology department; among the remaining hospitals, 33.0% were affiliated with clinical departments, 3.2% with logistics departments, and 3.2% with other departments. This organizational structure may constrain the department’s proactive role in multidisciplinary teams and its influence on clinical decision-making (Table 1).

### 3.2 Product structure

#### 3.2.1 Product availability and distribution

**Figure 1:**
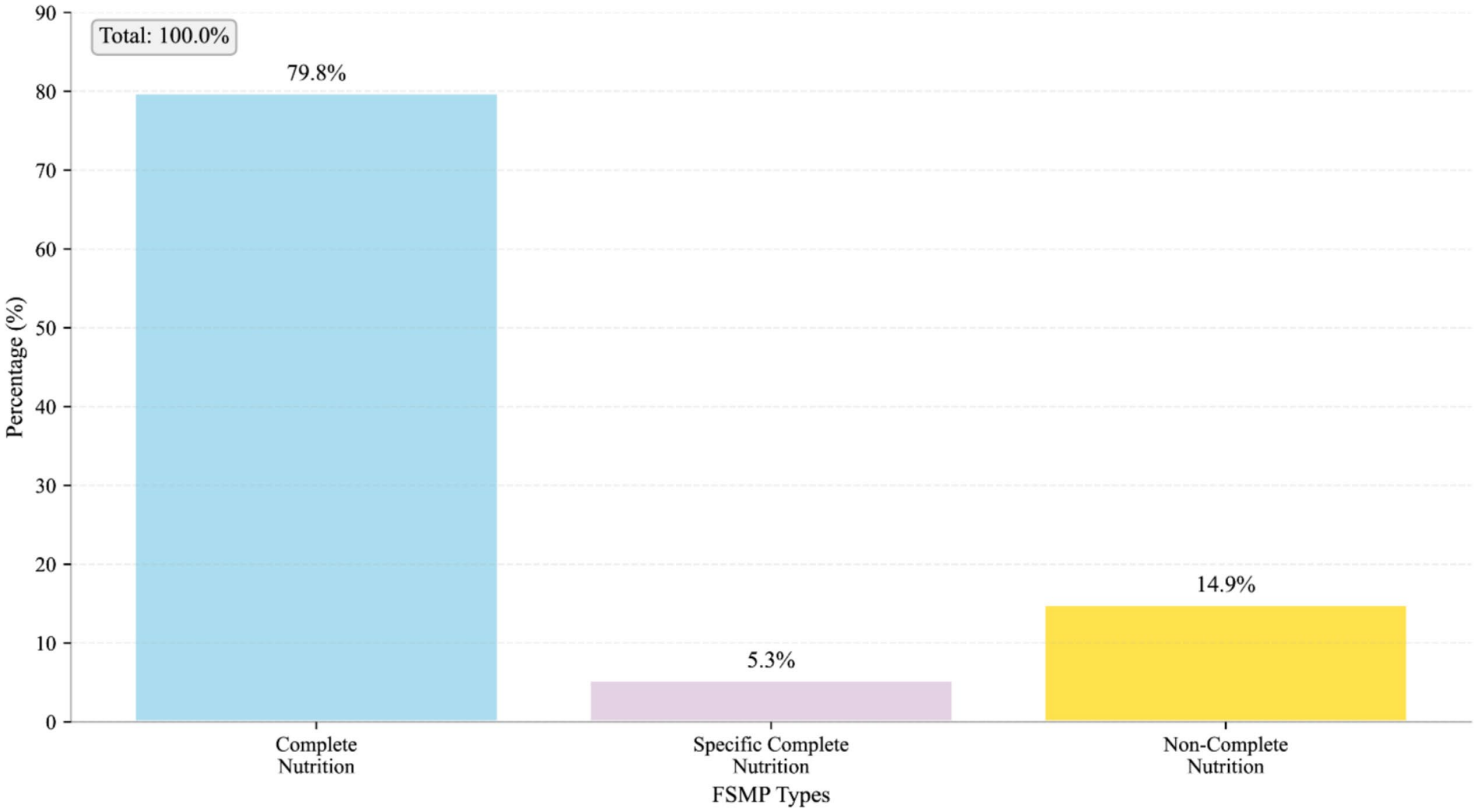
Types of FSMP currently provided by different hospitals.

**Figure 2:**
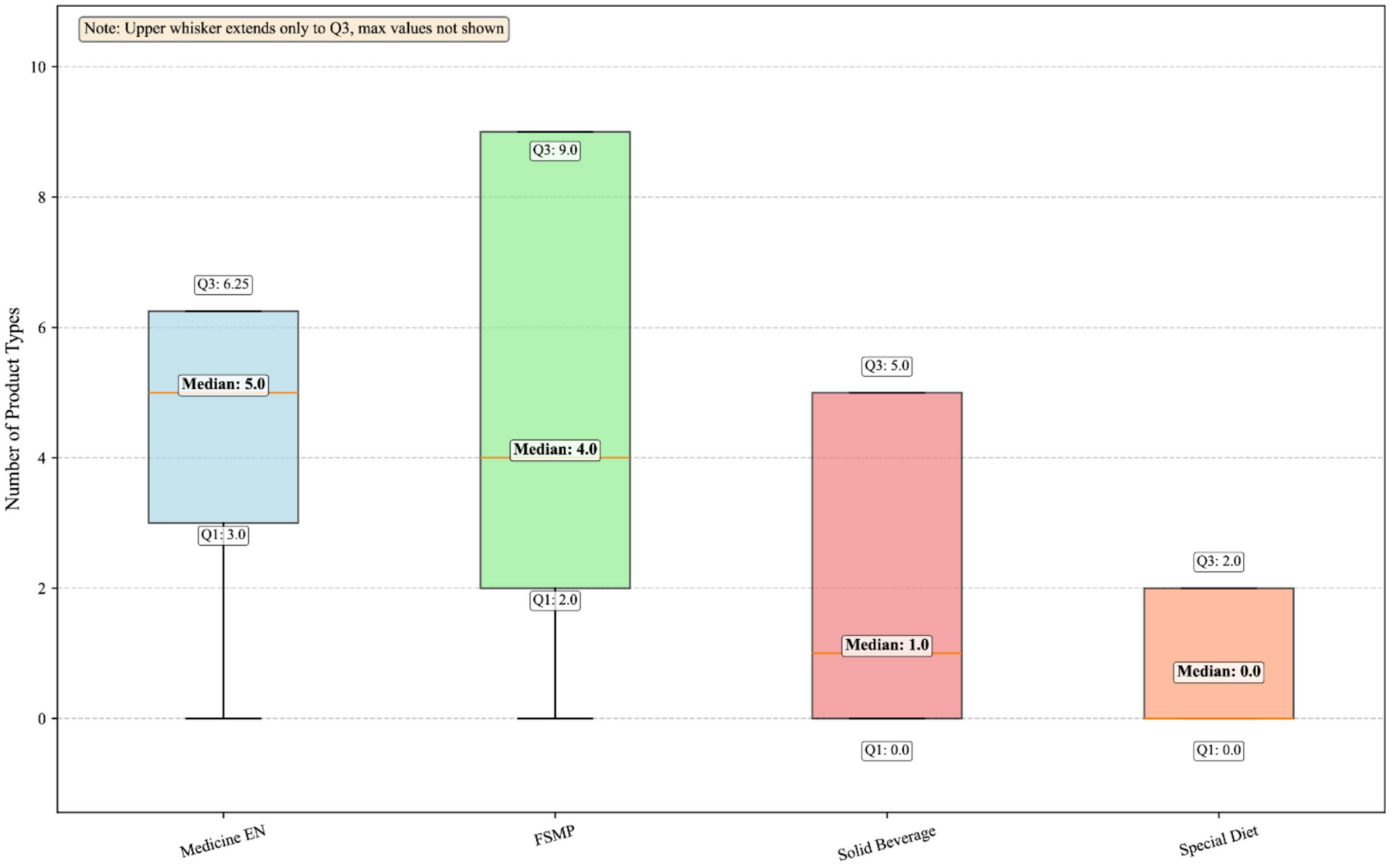
Categories and counts of available nutritional formulations.

The overall availability rate of FSMP in medical institutions across Jiangsu Province was 78.7% (*n* = 74; Table 1). In terms of product type distribution, nutritionally complete formulas dominated the market, accounting for 79.8%, whereas disease-specific nutritionally complete formulas and nutritionally incomplete formula components accounted for only 5.3 and 14.9%, respectively (Figure 1). The median number of FSMP products available for in-hospital prescription was 4 (IQR: 2–9; range: 0–47). Compared to FSMP, hospitals stocked a slightly higher median number of drug-registered enteral nutrition preparations [5 (IQR: 3–6.25)]. In contrast, the availability of solid beverages and foods for special dietary uses was much lower, with median numbers of 1 and 0 product, respectively (Figure 2).

#### 3.2.2 FSMP management system

**Figure 3:**
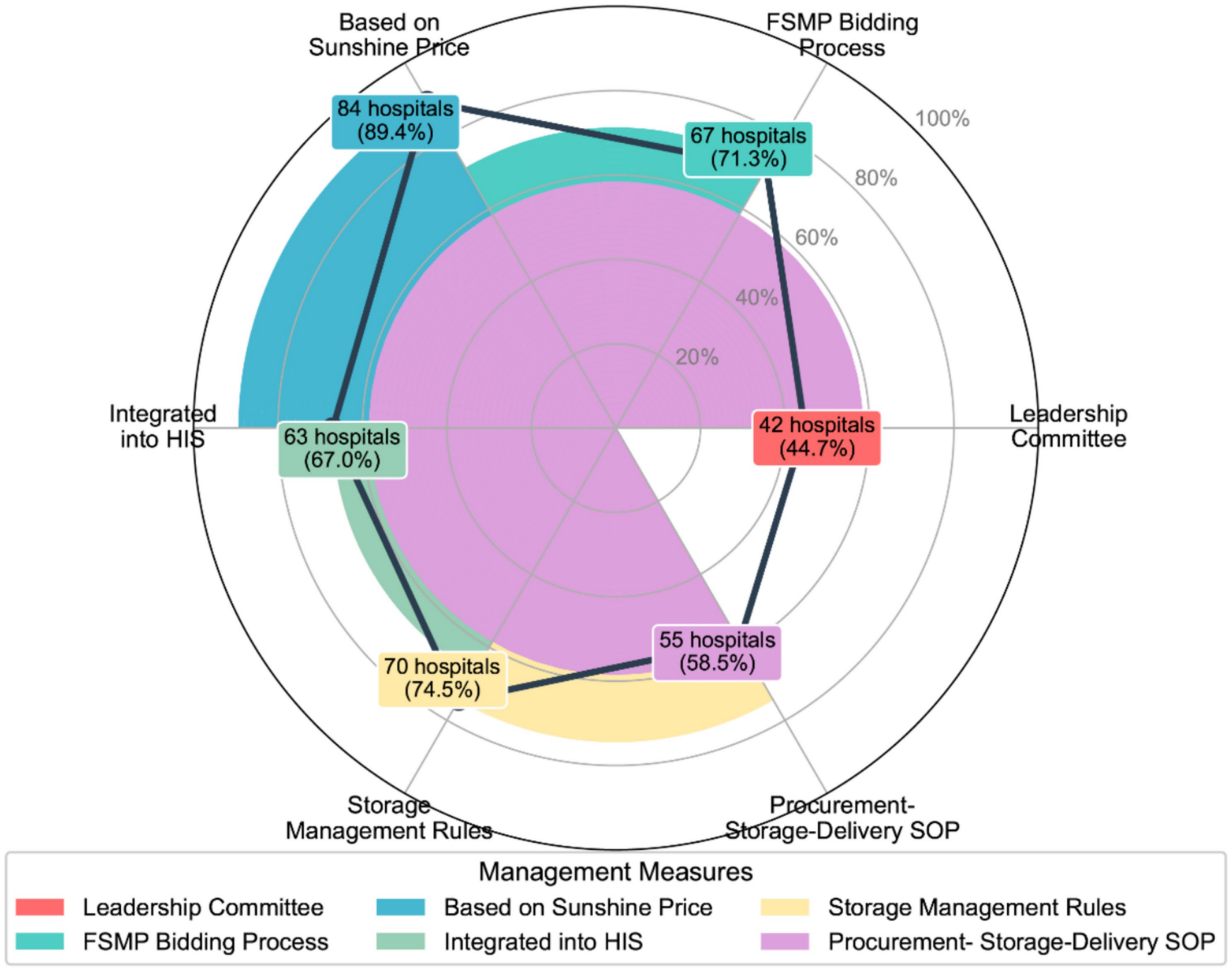
Radar chart analysis of the management system components.

**Figure 4:**
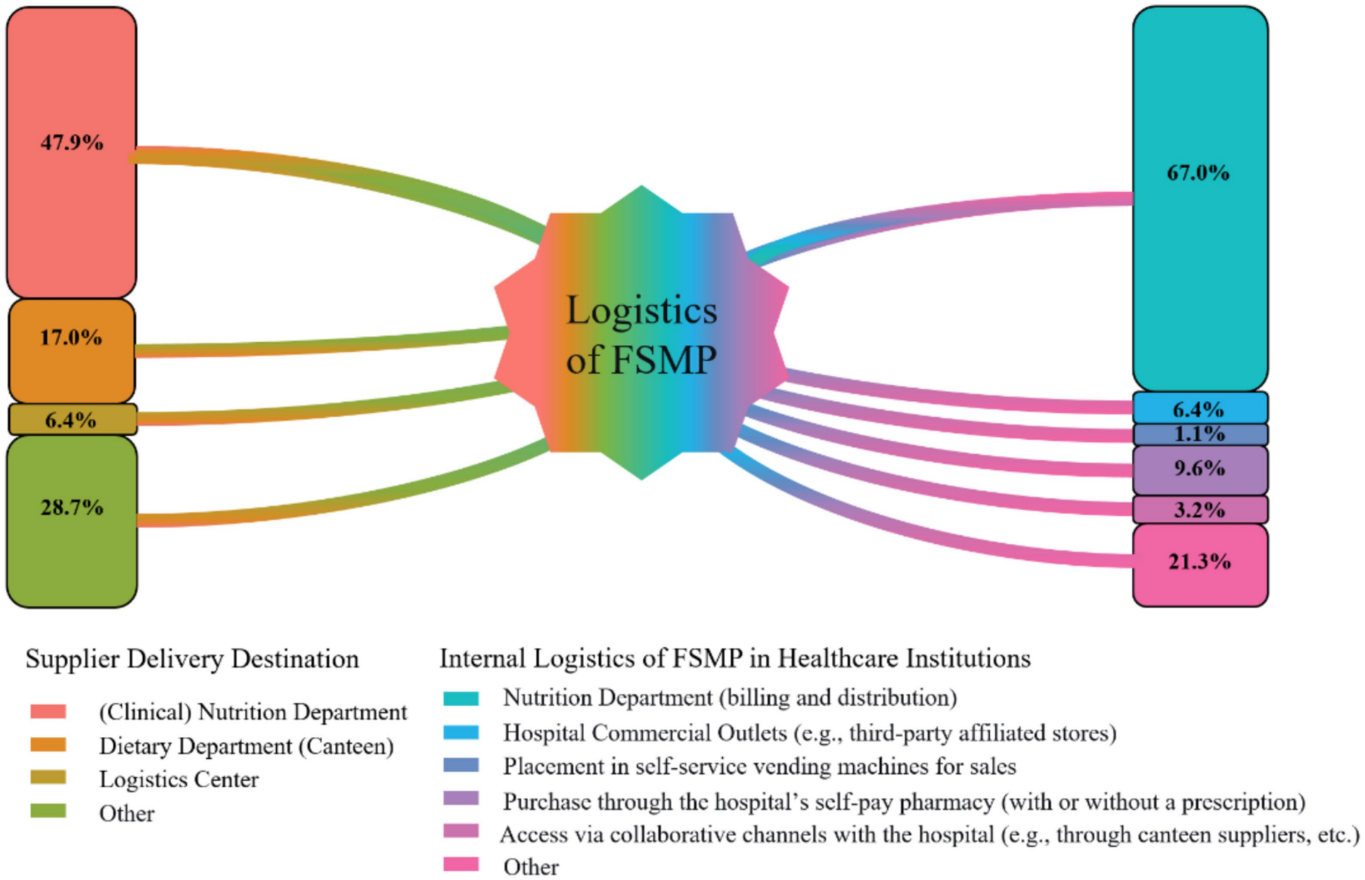
Flowchart of FSMP intra-hospital circulation pathways.

A standardized FSMP management system involving a cross-departmental committee was present in 44.7% of hospitals. Such committees were often chaired by senior hospital administrators. In terms of procurement, 71.3% of hospitals claimed to have implemented standardized bidding or selection procedures. Furthermore, 67.0% of hospitals had integrated FSMP products into their Hospital Information Systems (HIS; Figure 3). Regarding internal logistics and distribution, the Department of Clinical Nutrition served as the receiving point in 47.9% of cases; however, logistics centers (17.0%), canteens (6.4%), and other departments (28.7%) also functioned as distribution entry points for FSMP (Figure 4).

### 3.3 Clinical use

#### 3.3.1 Clinical application of FSMP

Regarding prescription privileges, clinical physicians held the highest proportion (86.2%), followed by nutrition physicians (69.1%) and dietitians (28.7%). A system for regular post-discharge follow-up for patients using FSMP had been established in 48.9% of the hospitals. Furthermore, 55.3% of the institutions had implemented specific monitoring and reporting processes for adverse reactions associated with FSMP. In terms of dispensing modes, 64.9% of hospitals exclusively provided finished commercial products, 4.3% offered only preparation services, while 30.9% provided both services (Table 2).

**Table 2:** Profile of FSMP clinical application.

Characteristics	Category	Number of hospitals (n)	Composition ratio (%)
Currently, which department is responsible for managing the pharmaceutical-grade enteral nutrition preparations?	Pharmacy department	90	95.7
	Other	4	4.3
The trend in the proportion of patients using pharmaceutical-grade enteral nutrition preparations throughout the hospital	Rise	41	43.6
	Just	44	46.8
	Decline	9	9.6
The usage method of the FSMP in your hospital	Only preparation	4	4.3
	Only the finished products will be distributed	61	64.9
	Both of the above situations exist	29	30.9
What percentage of the total number of patients using enteral nutrition products in your hospital used the FSMP?	≤25%	79	84.0
	25–50%	12	60.6
	≥50%	3	3.2
The prescription authority of FSMP in your hospital	Clinical doctor	81	86.2
	Nutrition doctor	65	69.1
	Nutritionist	27	28.7
	Other	7	7.4
Is there a regular education and supervision system established for the physicians and nutritionists related to FSMP?	Yes	48	51.1
	No	46	48.9
Does there exist a follow-up and monitoring management system for the clinical application effect of FSMP?	Yes	46	48.9
	No	48	51.1
Is there a regulatory system for adverse reactions to the clinical application of FSMP?	Yes	52	55.3
	No	42	44.7

#### 3.3.2 Cost and payment issues of FSMP

**Figure 5:**
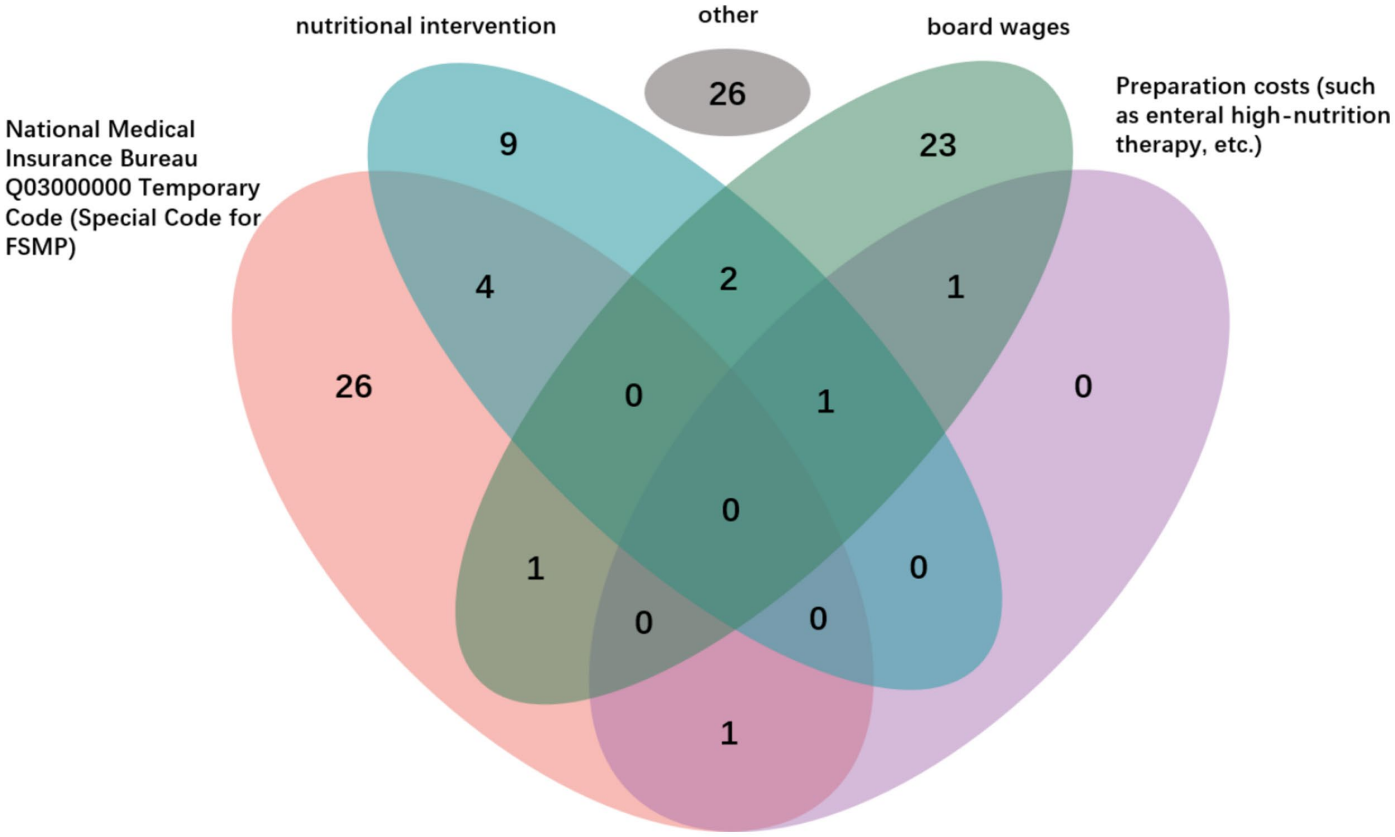
Composition of FSMP billing routes: A Venn diagram representation.

Regarding billing pathways, 29.8% of hospitals charged FSMP under the category of “meal fees.” Other billing methods included “nutrition intervention” (17.0%), “temporary FSMP codes from the Healthcare Security Administration” (34.0%), and “preparation fees” (3.2%; Figure 5). The proportion of hospitals allowing payment using personal medical insurance account balances was 38.3%. In terms of price transparency, 53.2% of hospitals publicly displayed the sales prices of FSMP. Fewer than one-third of hospitals (29.8%) incorporated appropriate FSMP use into departmental or staff performance appraisals. Even fewer (12.8%) had established specific incentive mechanisms and evaluations for FSMP application (Table 3).

**Table 3:** Financial mechanisms and related information.

Item	Yes (*n* = 94)	No (*n* = 94)
Is there a committee related to the use and management of FSMP that is led by an institute-level leader?	41(43.6)	53(56.4)
Are there corresponding FSMP bidding, selection or procurement procedures?	66(70.2)	28(29.8)
Does the FSMP procurement follow the prices listed on the Sunshine website?	83(88.3)	11(11.7)
Are there any regulations and procedures related to the inbound/outbound and storage management of FSMP?	69(73.4)	25(26.6)
Is there a system for establishing procurement - storage - distribution - receipt procedures (SOP)?	54(57.4)	40(42.6)

### 3.4 Analysis of heterogeneity in FSMP management across medical institutions with different characteristics

To further investigate the disparities in FSMP management and application, this study conducted a subgroup analysis based on the hospital’s geographic location, ownership type, tier, and category. Analysis of regional differences indicated that hospitals in Southern Jiangsu offered a greater variety of both FSMP and pharmaceutical-enteral nutrition products compared to those in Northern Jiangsu (*p* < 0.05). Specifically, the proportion of hospitals offering a high variety of FSMPs (≥4 types) was 59.0% in the southern region compared to 52.0% in the northern region (*p* = 29.983), Similarly, the proportion of hospitals with a high variety of pharmaceutical-grade enteral nutrition preparations (≥5 types) was significantly higher in the south at 79.5% versus 32.0% in the north (*χ^2^* = 27.872). Analysis of institutional differences revealed significant disparities in delivery locations, with private hospitals having a higher proportion of direct supplier delivery to the Nutrition Department (47.7% in public vs. 50.0% in private) and a notably higher rate of delivery to the Logistics Center (14.0% in public vs. 50.0% in private; *χ^2^* = 8.665). In contrast, public hospitals were more likely to have established standardized bidding processes (73.3% vs. 37.5%; *χ^2^* = 4.474) and adverse reaction monitoring systems (59.3% vs. 12.5%; *χ^2^* = 6.486). Furthermore, regarding FSMP utilization modes, public hospitals showed a stronger tendency to provide both compounding services and ready-to-use product distribution (32.8% vs. 12.5%), while private hospitals more frequently focused on a single service model, with a higher proportion offering compounding only (2.3% in public vs. 25.0% in private; *χ^2^* = 9.805). Results regarding hospital tier differences showed that tertiary hospitals had a higher rate of establishing an FSMP Management Committee led by senior administration (47.1% vs. 11.1%; *χ^2^* = 4.276), a greater proportion perceiving policies as facilitative (63.5% vs. 11.1%; *χ^2^* = 9.211), and a differing outlook on FSMP development prospects. While 41.2% of tertiary and 44.4% of secondary hospitals were optimistic, a higher percentage of tertiary hospitals held a neutral outlook (55.3% vs. 22.2%), and a greater proportion of secondary hospitals were pessimistic (3.5% in tertiary vs. 33.3% in secondary; *χ^2^* = 13.054). Finally, analysis by hospital category revealed that general hospitals exhibited a significantly higher proportion of institutions with ≥5 types of pharmaceutical-grade enteral nutrition preparations compared to non-general hospitals (60.2% vs. 33.3%; *χ^2^* = 26.99). Regarding FSMP utilization as a percentage of total enteral nutrition, general hospitals more commonly reported usage at ≤50% (90.4% vs. 76.1%; *χ^2^* = 33.885). Location of the FSMP warehouse also differed significantly; general hospitals were more likely to locate their FSMP warehouse within the Clinical Nutrition Department (64.4% vs. 38.1%), whereas specialty hospitals more frequently used the Logistics Center (4.1% vs. 23.8%) or Dietary Department (4.1% vs. 23.8%; *χ^2^* = 17.946; Table 4). All reported differences were statistically significant with *p* < 0.05.

**Table 4:** Differences in key FSMP management and application variables across different hospital types.

Projects with significant differences	Grouping		Statistical value (χ^2^)	Cramer’s V	*p*-value
	%(n)				
	The southern region of Jiangsu Province	The northern region of Jiangsu Province			
The current number of FSMP varieties available within the hospital (≥4 types)	59.0(*n* = 26)	52.0(*n* = 26)	29.983	0.565	0.038
The current number of product types of pharmaceutical-grade enteral nutrition preparations available in the hospital (≥5 types)	79.5(*n* = 35)	32.0(*n* = 16)	27.872	0.545	0.009
	Public hospital	Private hospital			
The location where the supplier delivers the FSMP to your hospital					
(Clinical) Nutrition Department	47.7(*n* = 41)	50.0(*n* = 4)	8.665	0.304	0.034
Dietary Department (Canteen)	7.0(*n* = 6)	0.0(*n* = 0)			
Logistics Center	14.0(*n* = 12)	50.0(*n* = 4)			
Other	31.4(*n* = 27)	0.0(*n* = 0)			
Standardized procedures for FSMP bidding, selection, and procurement are in place within the hospital.	73.3(*n* = 63)	37.5(*n* = 3)	4.474	0.218	0.034
FSMP Utilization Modes within Hospitals					
Compounding only	2.3(*n* = 2)	25(*n* = 2)	9.805	0.323	0.007
Distribution of ready-to-use products only	65.1(*n* = 56)	62.5(*n* = 5)			
Both modes coexist	32.8(*n* = 28)	12.5(*n* = 1)			
A regulatory mechanism for monitoring adverse reactions in FSMP clinical application is in place within the hospital.	59.3(*n* = 51)	12.5(*n* = 1)	6.486	0.263	0.011
	Grade III Hospital	Grade II Hospital			
An FSMP-specific committee, led by senior hospital administration, oversees its clinical use and management within the institution.	47.1(*n* = 40)	11.1(*n* = 1)	4.276	0.213	0.039
Policies have exerted a facilitative effect on the clinical use and administration of FSMP within the institution	63.5(*n* = 54)	11.1(*n* = 1)	9.211	0.313	0.002
Compared with pharmaceutical-grade enteral nutrition preparations, how should we view the development prospects of FSMP?					
Optimistic	41.29(*n* = 35)	44.4(*n* = 4)	13.054	0.373	0.001
Neutral	55.3(*n* = 47)	22.2(*n* = 2)			
Pessimism	3.5(*n* = 3)	33.3(*n* = 3)			
	General Hospital	Specialty Hospital			
Percentage of FSMP utilization in total enteral nutrition administrations					
≤50	90.4(*n* = 66)	76.1(*n* = 16)	33.885	0.600	0.037
≥50	9.6(*n* = 7)	23.9(*n* = 5)			
The current number of product types of pharmaceutical-grade enteral nutrition preparations available in the hospital (≥5 types)	60.2(*n* = 44)	33.3(*n* = 7)	26.99	0.536	<0.001
Where is the FSMP warehouse located within the hospital?					
Logistics Center	4.1(*n* = 3)	23.8(*n* = 5)	17.946	0.473	<0.001
(Clinical) Nutrition Department	64.4(*n* = 47)	38.1(*n* = 8)			
Dietary Department (Canteen)	4.1(*n* = 3)	23.8(*n* = 5)			
other	27.4(*n* = 20)	14.3(*n* = 3)			

### 3.5 Perceptions, attitudes, and future outlook of medical staff regarding FSMP

**Figure 6:**
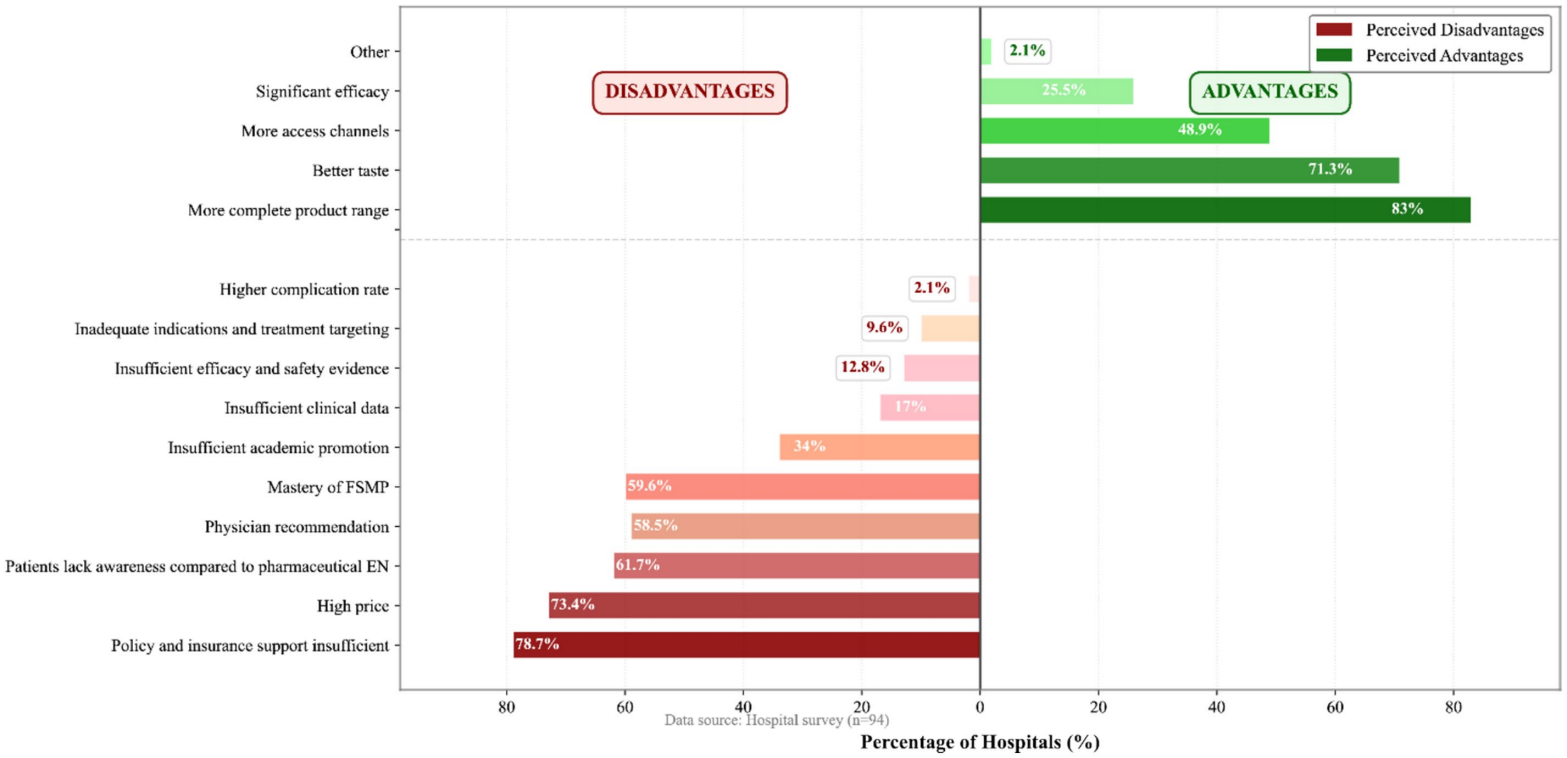
Clinicians’ views on the relative strengths and weaknesses of FSMP and drug-listed enteral nutrition products.

Hospital administrators and medical staff demonstrated a dualistic mindset characterized by recognition of product benefits versus concerns over implementation barriers. Regarding product attributes, respondents highly recognized the intrinsic advantages of FSMP, including “superior palatability and diversity of dosage forms” (71.3%) and “better ability to meet disease-specific nutritional requirements” (83.0%). However, “insufficient policy and medical insurance support” was identified as the paramount challenge (78.7%), followed closely by “inflated pricing of FSMP” (73.4%) and “low patient acceptance due to cognitive deficits” (61.7%; Figure 6).

Despite these hurdles, the outlook for FSMP development remains largely positive. The majority of respondents held a “neutral” (52.1%) or “cautiously optimistic” (41.5%) attitude, with only 6.4% expressing a “pessimistic” view regarding FSMP’s ability to compete with drug-registered enteral nutrition. In terms of the future competitive landscape, the prevailing view (59.6%) was that the market would “maintain the current status of drug-registered products while simultaneously expanding the variety and scale of FSMPs.” Meanwhile, 28.7% anticipated a “reduction in drug-registered products leading to their eventual integration into the FSMP registration system,” 9.6% predicted “long-term coexistence maintaining the status quo,” and only 2.1% held other views.

When queried about the primary challenges in an open-ended format, the qualitative analysis revealed that responses were highly concentrated on three core systemic barriers: Primarily manifested as high out-of-pocket costs for patients, lack of reimbursement pathways, and hospital-level restrictions due to cost-containment pressures under DRG/DIP reforms. Including ambiguous product identity, lack of unified billing codes, complex hospital entry procedures, and the inability to integrate FSMP into Hospital Information Systems (HIS). Characterized by insufficient attention and prescription motivation among clinicians, as well as poor patient compliance due to taste preferences or misconceptions about therapeutic value.

## 4 Discussion

This study, through systematic investigation and multi-dimensional data analysis, provides a comprehensive portrayal of the current landscape of FSMP application in medical institutions within Jiangsu Province—a province recognized as a national leader in the implementation and operationalization of FSMP-related policies. Despite this advantageous policy environment, the findings reveal persistent and significant challenges: a pronounced imbalance between high product penetration and growing clinical demand coexists with fragmented management systems, insufficient policy support, clinical application barriers, and limited cognitive awareness among healthcare professionals (20). These observations underscore the imperative for further refinement and maturation of relevant policies and practices.

Foods for Special Medical Purposes in Chinese medical institutions exhibit a profound paradox between high market penetration and low systemic integration. An institutional coverage rate of 78.7% has failed to translate automatically into standardized and sustainable clinical practice; instead, it has exposed a constellation of interconnected and mutually reinforcing systemic barriers. This contrast is particularly salient when compared with European and North American healthcare systems. We contend that the fundamental etiology of this dilemma resides in FSMP’s ambiguous institutional identity—positioned ambiguously at the interface between “food” and “medicine”—which precludes its seamless integration into existing hospital management infrastructures predominantly designed for pharmaceuticals and conventional medical consumables (18). By contrast, the European Union has established a rigorous regulatory framework through Regulation (EU) No 609/2013, which explicitly delineates the medical attributes of FSMP and facilitates their coherent integration into clinical pathways (21). The United States, while maintaining distinct regulatory pathways for “medical foods” and pharmaceuticals, mandates their utilization under medical supervision, thereby affirming their status as legitimate medical interventions (22).

Our findings indicate that FSMP has achieved quantitative parity with traditional drug-registered enteral nutrition preparations in terms of product diversity within hospital formularies. At the organizational level, however, over half of surveyed hospitals lack top-level design, coordination, and oversight mechanisms for FSMP governance, inevitably leading to ambiguous departmental responsibilities and ineffective policy implementation. In one-third of institutions, physicians are unable to prescribe FSMP with the same facility as conventional pharmaceuticals or drug-registered nutritional preparations through electronic information systems, substantially compromising clinical usability and management standardization. The internal circulation pathways of FSMP within hospitals exhibit considerable fragmentation, with multiple departments—including Nutrition, Logistics, and Hospital Food Services—potentially involved in product storage and distribution. Such fragmentation not only engenders management inefficiencies but also harbors latent risks to food safety and patient medication safety (9). In many Western medical centers, FSMP logistics are typically integrated into unified supply chain systems administered by Pharmacy or Clinical Nutrition departments, enabling comprehensive traceability from procurement to patient administration (23). In contrast, over half of Chinese hospitals have yet to establish FSMP management committees, institutionally substantiating this systemic imbalance.

The current state of FSMP clinical application reveals that nearly half of the hospitals operate with blind spots in monitoring potential safety risks, impeding timely detection and management of adverse events (24). Deficiencies in informatization not only compromise clinical convenience but also preclude timely data analysis, thereby frustrating data-driven quality improvement initiatives and outcome evaluations. Among these challenges, the financial positioning of FSMP within the healthcare payment system is particularly problematic, fundamentally misaligning with its essential attributes as a therapeutic product. This opacity in pricing further exacerbates information asymmetry during patient decision-making and product utilization. Payment barriers have emerged as among the most formidable obstacles to standardized FSMP application and widespread adoption. The pervasive absence of performance-linked incentives deprives clinicians and dietitians of sufficient economic motivation and institutional guidance to prescribe and utilize FSMP proactively and appropriately, thereby limiting further clinical penetration. The prevailing practice of billing FSMP under “meal fees” or requiring complete out-of-pocket payment epitomizes the failure of existing reimbursement systems to acknowledge FSMP’s medical value. With the nationwide implementation of Diagnosis-Related Groups (DRG) and Big Data Diagnosis-Intervention Packet (DIP) payment reforms, the absence of clear cost categorization and positioning of FSMP within total medical expenditures has subjected hospitals to heightened economic pressures and management uncertainties (25). Even in regions where temporary billing codes for FSMP have been established, inclusion in total hospitalization costs “consumes DRG quotas,” leading hospitals and clinicians to “exercise caution in utilization.” In the United Kingdom, the Advisory Committee on Borderline Substances (ACBS) has developed comprehensive lists authorizing general practitioners to prescribe FSMP for eligible patients, with costs reimbursed by the National Health Service (NHS) (5). Similarly, in Japan, specific enteral nutrition formulations are covered under the National Health Insurance (NHI). The experience from these countries demonstrates that formally acknowledging the therapeutic value of FSMP through payment policies not only improves clinical compliance but also, in the long term, contributes to reducing overall healthcare expenditure by preventing complications (26). In China, however, the absence of a supportive payment policy has become the most significant bottleneck impeding the standardized and rational application of FSMP.

The institutional and economic constraints inevitably shape and perpetuate specific clinical perceptions and behavioral patterns. The mindset revealed by this study—a coexistence of rational acknowledgment and insufficient trust among healthcare professionals—cannot be simply attributed to a knowledge deficit (27). Rather, it constitutes an adaptive rational choice within the aforementioned distorted institutional environment. Clinicians recognize the value of FSMP as a product, yet they widely lack confidence in the systemic reliability required for its effective, safe, and economically unambiguous application (28). This trust deficit stems from three primary concerns: firstly, perceived insufficient evidence, where physicians feel that compared to conventional pharmaceuticals, the data supporting FSMP efficacy for specific patient populations is not robust enough; secondly, ambiguous policies, encompassing uncertainty about billing procedures, reimbursement eligibility, and fear of triggering patient complaints; and thirdly, inadequate training on the standardized use of FSMP. Consequently, clinicians exhibit extreme caution in prescribing FSMP. This is not a reflection of conservatism but represents a form of rational self-protection within the current imperfect system.

Critically, the heterogeneity analysis of this study indicates that the severity and manifestations of this systemic syndrome are not uniformly distributed. Instead, they are profoundly modulated by specific contextual variables of the healthcare institutions, painting a picture of context-dependent challenges. Disparities in regional economic development amplify inequalities in resource accessibility; institutional ownership (public vs. private) fosters two distinct managerial paradigms prioritizing either regulatory compliance or operational efficiency; and hospital scale determines the threshold capacity for adopting and managing complex innovations. Furthermore, the needs and management models differ substantially between specialized hospitals and general hospitals. This evidence strongly substantiates that the FSMP predicament constitutes a highly systematic and complex issue. It presents with varying facets in different environments, demonstrating that any one-size-fits-all solution is destined to be ineffective.

Based on this systemic diagnosis, sporadic improvement measures targeting isolated symptoms are destined to yield limited results. We therefore propose an integrated governance framework centered on “restructuring institutional identity as the precursor, followed by four-layer synergistic interventions.” First, regarding institutional identity, it is recommended that the National Health Commission and the National Healthcare Security Administration jointly create an independent billing category for FSMP, such as “Medical Nutrition Therapy,” formally acknowledging it as an integral part of medical practice. This is the logical prerequisite for breaking all subsequent deadlocks (17). Second, payment system reform must establish a multi-source financing model encompassing “basic medical insurance + personal health accounts + commercial insurance + special fiscal funds” (22). In the short term, the conflict under DRG/DIP must be resolved immediately by explicitly stipulating that FSMP costs are “excluded from uploads to the insurance system and from total hospitalization budgets and DRG/DIP settlements,” enabling separate payment (17). In the medium term, there should be a proactive response to the widespread call for allowing “payment via personal health savings accounts.” Pilot programs for local insurance reimbursement should be initiated, prioritizing clinically essential and evidence-specific complete nutritional formulas (e.g., for oncology, chronic kidney disease), thereby eliminating financial concerns for hospitals and physicians. Third, within hospitals, fragmented management must be decisively terminated through strong administrative mandates (29). Official guidelines should stipulate the consolidation of full-process management authority for FSMP—from selection, procurement, and storage to prescription review and distribution—under a single department, either the Department of Clinical Nutrition or the Pharmacy Department. Leveraging their professional expertise, this department would establish Standard Operating Procedures (SOPs) to achieve closed-loop management and quality control from supplier to patient. Concurrently, key performance indicators such as “rate of FSMP management committee establishment” and “rate of standardized application” should be incorporated into hospital accreditation and public hospital performance evaluation systems, creating a powerful external driving force. Finally, guided by the heterogeneity findings of this study, we propose a “Stratified and Categorized” precision governance system:

1. Regional Stratification: Establish regional equity compensation mechanisms, providing targeted support to economically underdeveloped areas.
2. Ownership Categorization: Implement differentiated guidance strategies respecting the distinct institutional logics of public and private hospitals.
3. Hospital Tier Stratification: Promote capacity-building programs tailored to hospital grades, with enhanced support for secondary hospitals.
4. Hospital Type Categorization: Encourage the development of integrated protocols based on clinical pathways, achieving deep fusion between FSMP management and specialty-specific care models.

These four interventions form an interdependent and synergistic organic whole. A four-pronged strategy is essential: first, redefine its legal status (the foundation); second, reform reimbursement (the key); third, strengthen institutional management (the safeguard); and fourth, reshape clinical cognition (the root). This holistic approach is the only way to liberate FSMP from institutional ambiguities and realize its benefits for patients and health systems. Beyond FSMP, our findings offer a valuable reference for governing other boundary-spanning medical innovations. The overarching aim is to ensure that FSMP—a valuable clinical tool—can be deployed safely, in a standardized manner, and sustainably, thereby delivering reliable benefits to patients.

## 5 Originality and limitations

To the best of our knowledge, this study represents the first cross-sectional investigation to systematically examine the entire continuum of FSMP—spanning policy design, clinical application, and standardized management—within medical institutions in Jiangsu Province, China. By leveraging real-world data on FSMP utilization, this study elucidates critical deficiencies in both clinical practice and managerial frameworks, thereby furnishing empirical evidence to inform the formulation of more targeted policy interventions in Jiangsu Province and beyond.

Despite these contributions, this study has several limitations that should be acknowledged. First, the sample was drawn from Jiangsu Province, a developed region in eastern China. While Jiangsu is representative in terms of economic development and healthcare resources, caution is warranted when extrapolating the findings to central and western regions with relatively lower socioeconomic levels. Future research should expand the scope to a national scale. Second, the cross-sectional survey design captures the status quo at a specific point in time and cannot dynamically reveal causal relationships in the evolution of FSMP management. Third, although rigorous quality control measures were implemented during questionnaire administration, the data may still contain certain biases. Subsequent studies could integrate methods such as field observations and in-depth interviews, and cross-validate findings with objective data extracted from Hospital Information Systems (HIS), to achieve a more profound and accurate understanding. Finally, while the subgroup analysis in this study revealed significant disparities, the exploration of the underlying mechanisms behind these differences warrants further investigation.

## 6 Conclusion

This cross-sectional survey of 94 healthcare institutions in Jiangsu Province confirms the significant paradox of “high penetration yet low management standardization” regarding the application of Food for Special Medical Purposes (FSMP) in Chinese medical institutions. Despite achieving a product coverage rate of 78.7%, substantial issues of non-standardization and fragmentation persist in the management system, intra-hospital circulation pathways, and payment mechanisms. Furthermore, this study provides the first quantitative evidence of the structural heterogeneity in FSMP management across different regions, hospital tiers, and ownership types, revealing the profound influence of resource endowment and institutional environments on management models.

The primary contribution of this study lies in constructing an analytical framework from an integrated perspective, identifying “ambiguous institutional identity” and the “absence of medical insurance coverage” as the core bottlenecks hindering the standardized application of FSMP. Accordingly, it proposes a systematic governance strategy based on “identity restructuring, payment reform, and system reconstruction.” However, this study is limited by its reliance on cross-sectional data from a single province, which may not fully represent the situation in less developed regions of central and western China, and by the lack of longitudinal data to track the long-term effects of policy interventions.

Looking ahead, it is recommended that policymakers prioritize establishing an independent medical billing code for FSMP and explore diversified payment and compensation mechanisms. Future research should expand to a national scale, investigate the long-term impact of DRG/DIP payment reforms on clinical nutrition practice, and conduct prospective studies to evaluate the specific contributions of different management models to patient clinical outcomes and health economic benefits.

## Data Availability

The raw data supporting the conclusions of this article will be made available by the authors, without undue reservation.
